# Supplementary material for: Resolving the full spectrum of human genome variation using Linked-Reads
Source: Genome Res. 2019 Apr;29(4):635–45. doi: 10.1101/gr.234443.118 (PMC6442396; doi:10.1101/gr.234443.118)
Supplement: Supplemental Material [file supp_gr.234443.118_Supplemental_longranger-2.2.zip › longranger-2.2.tar.bz2!/lr2.2-public/lib/stan/cmdstan/stan/doc/api/html/classstan_1_1mcmc_1_1covar__adaptation.html]

Stan: stan::mcmc::covar\_adaptation Class Reference


|  |  |
| --- | --- |
|  | Stan  2.5.0  probability, sampling & optimization |


- Main Page
- Related Pages
- Namespaces
- Classes
- Files

- Class List
- Class Index
- Class Hierarchy
- Class Members

All Classes Namespaces Files Functions Variables Typedefs Enumerations Enumerator Friends Macros Pages

- stan
- mcmc
- covar\_adaptation

Public Member Functions |
Protected Attributes |
List of all members

stan::mcmc::covar\_adaptation Class Reference

`#include <covar_adaptation.hpp>`

Inheritance diagram for stan::mcmc::covar\_adaptation:

|  |  |
| --- | --- |
| Public Member Functions | |
|  | covar\_adaptation (int n) |
|  | |
| bool | learn\_covariance (Eigen::MatrixXd &covar, const Eigen::VectorXd &q) |
|  | |
| Public Member Functions inherited from stan::mcmc::windowed\_adaptation | |
|  | windowed\_adaptation (std::string name) |
|  | |
| void | restart () |
|  | |
| void | set\_window\_params (unsigned int num\_warmup, unsigned int init\_buffer, unsigned int term\_buffer, unsigned int base\_window, std::ostream \*e=0) |
|  | |
| bool | adaptation\_window () |
|  | |
| bool | end\_adaptation\_window () |
|  | |
| void | compute\_next\_window () |
|  | |

|  |  |
| --- | --- |
| Protected Attributes | |
| prob::welford\_covar\_estimator | estimator\_ |
|  | |
| Protected Attributes inherited from stan::mcmc::windowed\_adaptation | |
| std::string | estimator\_name\_ |
|  | |
| unsigned int | num\_warmup\_ |
|  | |
| unsigned int | adapt\_init\_buffer\_ |
|  | |
| unsigned int | adapt\_term\_buffer\_ |
|  | |
| unsigned int | adapt\_base\_window\_ |
|  | |
| unsigned int | adapt\_window\_counter\_ |
|  | |
| unsigned int | adapt\_next\_window\_ |
|  | |
| unsigned int | adapt\_window\_size\_ |
|  | |

## Detailed Description

Definition at line 14 of file covar\_adaptation.hpp.

## Constructor & Destructor Documentation

|  |  |  |  |  |  |
| --- | --- | --- | --- | --- | --- |
| stan::mcmc::covar\_adaptation::covar\_adaptation | ( | int | *n* | ) |  |

inline

Definition at line 18 of file covar\_adaptation.hpp.

## Member Function Documentation

|  |  |  |  |
| --- | --- | --- | --- |
| bool stan::mcmc::covar\_adaptation::learn\_covariance | ( | Eigen::MatrixXd & | *covar*, |
|  |  | const Eigen::VectorXd & | *q* |
|  | ) |  |  |

inline

Definition at line 20 of file covar\_adaptation.hpp.

## Member Data Documentation

prob::welford\_covar\_estimator stan::mcmc::covar\_adaptation::estimator\_

protected

Definition at line 48 of file covar\_adaptation.hpp.

---

The documentation for this class was generated from the following file:

- src/stan/mcmc/covar\_adaptation.hpp


---

[ Stan Home Page ]

*© 2011–2014,
Stan Development Team.*
